# Optimizing adalimumab treatment in psoriasis with concomitant methotrexate (OPTIMAP): study protocol for a pragmatic, single-blinded, investigator-initiated randomized controlled trial

**DOI:** 10.1186/s13063-017-1777-y

**Published:** 2017-02-02

**Authors:** Celine Busard, Stef Menting, Sun-Jine van Bezooijen, Juul van den Reek, Barbara Hutten, Errol Prens, Elke de Jong, Martijn van Doorn, Phyllis Spuls

**Affiliations:** 1 Department of Dermatology, Academic Medical Center, Amsterdam, The Netherlands; 2 Department of Dermatology, Erasmus University Medical Center, Rotterdam, The Netherlands; 3 Department of Dermatology, Radboud University Medical Center, Nijmegen, The Netherlands; 4 Department of Clinical Epidemiology, Biostatistics and Bioinformatics, Academic Medical Center, Amsterdam, The Netherlands

**Keywords:** Multicenter, Randomized controlled trial, Pragmatic, Psoriasis, Combination therapy

## Abstract

**Background:**

The introduction of anti-tumor necrosis factor medications has revolutionized the treatment of psoriasis with achievement of treatment goals (Psoriasis Area and Severity Index score 75, remission) that are not usually met with conventional systemics. Nevertheless, some patients continue to experience persistent disease activity or treatment failure over time. Strategies to optimize treatment outcomes include the use of concomitant methotrexate, which has demonstrated beneficial effects on pharmacokinetics and treatment efficacy in psoriasis and other inflammatory diseases.

**Methods:**

This is an investigator-initiated, multicenter randomized controlled trial (RCT) designed to compare the combination treatment of adalimumab and methotrexate with adalimumab monotherapy in patients with psoriasis. The primary outcome is adalimumab drug survival at week 49. Other outcomes include improvement in disease severity and quality of life, tolerability, and safety. Moreover, anti-adalimumab antibodies and adalimumab serum concentrations will be measured and correlations between genotypes and clinical outcomes will be assessed. Patient recruitment started in March 2014. Up to now, 36 patients have been randomized. Many more patients have been (pre)screened. A total of 93 patients is desired to meet an adequate sample size. In our experience, the main limitation for recruitment is prior adalimumab therapy and intolerability or toxicity for methotrexate in the past.

**Discussion:**

OPTIMAP is the first RCT to examine combination therapy with adalimumab and methotrexate in a psoriasis population. With data derived from this study we expect to provide valuable clinical data on long-term treatment outcomes. These data will be supported by assessment of the impact of concomitant methotrexate on adalimumab pharmacokinetics. Furthermore, the influence of several single nucleotide polymorphisms on adalimumab response will be analyzed in order to support the development of a more personalized approach for this targeted therapy.

**Trial registration:**

NTR4499. Registered on 7 April 2014.

**Electronic supplementary material:**

The online version of this article (doi:10.1186/s13063-017-1777-y) contains supplementary material, which is available to authorized users.

## Background

Adalimumab has been shown to be highly valued by patients with psoriasis due to its profound improvements in disease severity and its favorable safety profile [1, 2]. Although its introduction (together with other anti-tumor necrosis factor (TNF) medications) has majorly advanced psoriasis care, some patients experience persistent disease activity (primary non-responders), treatment failure over time (secondary non-responders), or side effects [3–5]. Several factors have been identified to play a role in primary and secondary non-response to anti-TNFs, including pharmacokinetic factors such as the formation of anti-drug antibodies (immunogenicity) and inter-individual variation in serum drug concentrations as well as pharmacogenetic factors such as the absence or presence of certain single nucleotide polymorphisms (SNPs) affecting drug metabolization [6, 7].

When anti-drug antibodies are formed in patients treated with an anti-TNFα, clearance of the biologic can, to a certain extent, be accelerated depending on the concentration of the anti-drug antibodies [8]. Moreover, anti-drug antibodies can be functionally neutralizing, thereby directly affecting treatment efficacy [9]. Multiple studies observed an association between the formation of anti-adalimumab antibodies, reduced serum levels, and diminished clinical response in psoriasis and other chronic inflammatory diseases [3, 10–13]. In rheumatoid arthritis (RA) and Crohn's disease, concomitant use of methotrexate (MTX) during treatment with certain TNFα inhibitors (adalimumab, infliximab, and golimumab) has been demonstrated to decrease immunogenicity and significantly reduce clearance, resulting in higher systemic exposure and enhanced clinical efficacy [11, 14–18]. Therefore, the use of combination therapy may be beneficial for successful long-term adalimumab treatment. In addition, combination therapy may enable dose reductions of individual agents, thereby decreasing toxicity and improving tolerability and compliance [19]. By targeting unregulated increased cytokine levels associated with inflammatory comorbid conditions, it is hypothesized that combination therapy may also provide a broader benefit to the patient by reducing the risk of, for example, cardiovascular events [20].

On the other hand, combination therapy may theoretically convey an increased risk for serious infections and malignancies.

Currently available evidence on anti-TNFα therapy with MTX in psoriasis is limited to two randomized controlled trials (RCTs) on etanercept with MTX [19, 21, 22] and a few observational studies and case series on other different anti-TNFα agents with MTX [23–25]. The two RCTs on etanercept and MTX provided promising results with superior efficacy of etanercept with MTX compared to etanercept monotherapy. RCTs investigating combined treatment with adalimumab and MTX are lacking [19, 26].

In order to investigate whether adalimumab treatment can be optimized by using concomitant MTX, long-term clinical and pharmacokinetic data on the use of adalimumab in combination with MTX are desired. Additionally, as several polymorphisms have been identified as potential predictors for anti-TNF therapy in psoriasis (e.g., TNFR1B, TNFAIP3, IL12B/IL23R) [6, 27] and other chronic inflammatory diseases (e.g., FcGR and ATG16L1) [28, 29], it will be valuable to detect genetic factors associated with response to adalimumab in order to support personalized care.

### Aims and objectives

The aims and objectives of this trial are:

- To gain long-term RCT data on the efficacy and safety of adalimumab combined with MTX compared to adalimumab monotherapy
- To assess the impact of concomitant MTX on adalimumab immunogenicity and serum concentrations
- To test appropriate candidate genes and correlate genotypes with trial outcomes

## Methods

This is a multicenter RCT reported according to the Standard Protocol Items: Recommendations for Interventional Trials (SPIRIT) guidelines (see Table 1 (SPIRIT table) and Additional file 1 (SPIRIT checklist)). The trial was granted ethics approval by the Academic Medical Center research ethics committee (METC 2013_346). The trial is registered at The Netherlands National Trial Register (trial number: NTR4499) and in the European Clinical Trials Database (EudraCT number: 2013-004918-18). All participants will sign informed consent before participation. The study is being conducted according to the principles of the Declaration of Helsinki and in accordance with the Medical Research Involving Human Subjects Act (WMO) and other relevant guidelines, regulations, and acts.

**Table 1:** Timeline of the study according to the SPIRIT guidelines

Time	Screening	Week −2	Week −1	Week 0	Week 3	Week 5	Every 12 weeks from week 13 up to week 145	Early termination
Enrollment								
Informed consent	X							
Demography, medical history	X							
Vital signs and full physical examination	X			X		X	X	X
Psoriasis Area and Severity Index (PASI)	X			X		X	X	X
Eligibility criteria	X							
Laboratory assessments	X		X	X	X	X	X	X
Chest X-ray, Mantoux test, and interferon-gamma release assay (IGRA)	X							
Randomization	X							
Intervention								
Adalimumab (Humira) + MTX		X (MTX)	X (MTX)	X (Humira + MTX)				
Humira monotherapy				X (Humira)				
Assessments								
Laboratory assessments	X		X	X	X	X	X	X
PASI	X			X		X	X	X
Physician global assessment	X			X		X	X	X
Patient global assessment	X			X		X	X	X
Impact on quality of life (Skindex 29 and Dermatology Life Quality Index (DLQI))	X			X		X	X	X
Prior or concomitant therapy	X			X		X	X	X
Adverse events	X			X		X	X	X
Drug adherence				X		X	X	X

### Participants

Patients will be recruited from the outpatient clinics of the Departments of Dermatology of the Academic Medical Center (AMC) Amsterdam, Erasmus University Medical Center (EMC) Rotterdam, and the Radboud University Medical Center (RUMC) Nijmegen. Moreover, other dermatologists will be contacted to recruit and refer eligible patients to the participating centers. Participants must meet the inclusion criteria and none of the exclusion criteria (Table 2) in order to participate. These criteria will be assessed at the screening visit. Potential participants who are deemed ineligible at screening will be allowed a second screening visit if the reason for ineligibility is a temporary status (e.g., latent tuberculosis).

**Table 2:** Eligibility criteria

Inclusion	Exclusion
≥18 years Diagnosis of moderate to severe plaque psoriasis (PASI ≥8) Adalimumab naïve Candidate for biologic therapy Willing and able to use adequate contraceptives during the study	History of significant MTX toxicity, intolerability, or contraindication Known liver or kidney malfunction Alcohol abuse Bone marrow hypoplasia, leukocytopenia, thrombocytopenia, or significant anemia Known severe or chronic infections like tuberculosis or HIV Ulcers in the oral cavity or known active ulcers in digestive tract Pregnant or nursing women Need for live vaccinations Use of other immunosuppressive medication (e.g., prednisone, mycophenolate mofetil (Cellcept), cyclosporine (Neoral), sirolimus (Rapamune), systemic tacrolimus (Prograf))

### Interventions

All patients receive adalimumab 40 mg subcutaneously every other week starting 1 week after a loading dose of 80 mg and will be randomized 1:1 to receive either oral MTX 10 mg weekly (combination group) or no addition of MTX (monotherapy group). MTX therapy will be initiated 2 weeks prior to adalimumab therapy, and administration will be followed by folic acid 5 mg 24 hours after MTX intake (see Table 1).

In case of MTX toxicity (e.g., liver toxicity or leukopenia) or intolerability, dosing can be paused (for a maximum of 2 weeks up to four times during the entire study), or the dose can be adjusted to 7.5 mg. Moreover, patients are allowed to switch from oral to subcutaneous administration. In case of adalimumab toxicity or intolerability, dosing can be paused (for a maximum of 2 weeks up to four times during the entire study).

Throughout the study, no systemic anti-psoriatic drugs are allowed for treatment other than the study medication (Table 3). If medically necessary (i.e., to control intolerable psoriasis activity), rescue treatment with topical corticosteroids, vitamin D derivates (calcipotriol/betamethasone or calcitriol), or calcineurin inhibitors may be provided to study patients at the discretion of the investigator after baseline and through week 145 (end of study) (Table 4).

**Table 3:** Wash-out periods

Therapy	Wash-out period
Topical therapy	2 weeks
Phototherapy	2 weeks
Conventional systemic therapy/etanercept	4 weeks
Infliximab/ustekinumab	6 weeks

**Table 4:** Allowed escape medication

Scalp/palms/soles	Low or high potency corticosteroids, calcitriol/calcipotriol, or a combination
Face and body	Low potency corticosteroids, calcitriol/calcipotriol, or topical tacrolimus 0.1% or 0.03%
inverse psoriasis	Topical tacrolimus 0.1% or 0.03%

### Randomization and blinding

Consecutive patients will be prospectively enrolled and randomly assigned if eligible to either the intervention (adalimumab with MTX) or control (adalimumab monotherapy) group after obtaining informed consent. Each consecutive patient will be assigned a randomization number according to a computer-generated randomization list (ALEA) using random block sizes of 2, 4, 6, and 8 to ensure allocation concealment. Randomization is stratified for TNFα-blocker exposure status to achieve balance with regard to prior TNFα-blocker exposure in the study population.

This is an observer-blinded study. The observer (outcome assessor) will perform clinical outcome assessments of disease severity (Psoriasis Area and Severity Index (PASI) and investigator global assessment (IGA)) at each study visit. The clinician performs all other study procedures and is not blinded. Both clinician and participant know the treatment allocation; as such, no special measures are required to allow for breaking of treatment codes. However, treatment allocation will not be revealed to the recruiting physician until participants’ details and key stratification variables have been irrevocably entered onto the web-based randomization site.

### Endpoints

The primary outcome is adalimumab drug survival (number of patients still on adalimumab treatment) at week 49.

Secondary outcomes are the following:

- Adalimumab drug survival (number of patients still on adalimumab treatment) at week 145
- Proportion of patients who reach treatment goals^1^ at week 13, week 25, week 49, and week 145
- Proportion of patients achieving PASI 75 at weeks 49 and 145
- Proportion of patients achieving IGA clear or almost clear at weeks 49 and 145
- Mean improvement in PASI at weeks 49 and 145
- Proportion of patients with PGA clear or almost clear at weeks 49 and 145
- Mean improvement in Dermatology Life Quality Index (DLQI) and Skindex at weeks 49 and 145
- Proportion of patients with (serious) adverse events at weeks 49 and 145
- Proportion of patients with changes in laboratory assessments at weeks 49 and 145
- Proportion of patients with (no, low, or high) levels of antibodies at weeks 49 and 145
- Median adalimumab trough concentrations (mg/L) at weeks 49 and 145
- Correlation between genetic polymorphisms and adalimumab response

### Procedures and assessments

Patients will visit the outpatient clinic at screening, baseline, week 5, week 13, and every 12 weeks thereafter until study completion (weeks 25, 37, 49, 61, 73, 85, 97, 109, 121, 133, 145) (Table 1). A variety of parameters will be collected during each visit to assess efficacy, including physician- (PASI/IGA (static; scale 0–4 [30])) and patient-reported (patient-reported global assessment (PGA static; scale 0–4)) outcomes. Quality of life assessment will be performed using Skindex and DLQI questionnaires. Safety will be assessed by evaluating the incidence of (serious) adverse events, obtaining a detailed medical history, thorough physical examination, vital signs, clinical laboratory testing, and urinalysis (including pregnancy tests for females of childbearing potential at screening). Concomitant medication and medical procedures will be collected from obtainment of informed consent up to end of study. Patients will receive a diary in which they will register the administration dates of adalimumab (and MTX in the combination group), any changes in their health status, and/or changes in concomitant medication used. The local investigator reviews the diary to determine drug adherence and the incidence and type of adverse events. An independent Data Safety Monitoring Board (DSMB) has been established to review efficacy and safety data periodically in an unblinded fashion.

### Laboratory testing

Blood samples will be collected at each visit (serum samples are collected just before administration of adalimumab (3-day window) to ensure accurate determination of serum through levels) to monitor drug safety, to determine immunogenicity against adalimumab, and to measure adalimumab serum through levels. Samples for serum preparation are kept at room temperature for 1–2 hours for coagulation, followed by centrifugation at 3000 RPM for 15 minutes at room temperature. Supernatant is collected, aliquoted, and stored at −20 °C until further use. Adalimumab serum through levels will be determined using a non-commercial enzyme-linked immunosorbent assay (ELISA, Sanquin, Amsterdam, The Netherlands). Detection of anti-adalimumab antibodies will be performed through a radioimmunoassay (Sanquin). The antibody test will be considered positive when the antibody concentration exceeds 12 AU/mL. Concentrations between 12 and 100 AU/mL will be considered low antibody titers; those above 100 AU/mL will be considered high antibody titers [3].

Additionally, a single blood sample will be collected at screening from which DNA will be collected and stored at −80 °C. As scientific interest in this field is currently increasing, DNA analysis will be performed based upon accumulating data acquired from (ongoing) pharmacogenetic studies.

### Justification of sample size

A total of 84 patients (randomized 1:1 to concomitant MTX or no MTX) will give the study at least 80% power at a 0.05 two-sided significance level using a two-sample chi-squared test to detect a difference of 28% in drug survival at week 49. We aim to enroll 93 patients to allow for an approximate 10% loss to follow-up. These calculations were performed using Nquery 6.0.2. Due to the lack of data in a psoriasis population, the expected clinically relevant difference in drug survival between both treatment groups was hypothesized based on studies performed in patients with RA. The prevalence of (clinically relevant) anti-drug antibody formation is estimated to be 45% in patients on adalimumab monotherapy (a similar percentage is found in patients with psoriasis [31] and around 17% in patients on adalimumab with low-dose (5–10 mg) MTX after 49 weeks [32]. A clear correlation between antibody formation and treatment failure (with subsequent treatment discontinuation) in patients on adalimumab has been demonstrated [31]. Based on these data, drug survival is estimated to be 83% (100 minus 17) for the experimental group and 55% (100 minus 45) for the control group after 49 weeks of follow-up.

### Statistical analysis

The primary analysis will be conducted on the intention-to-treat population, including all randomized participants in the groups to which they were randomized. A per-protocol population (excluding major protocol violations) will be used to check the robustness of the primary analyses. The safety population will consist of all patients receiving at least one dose of the study drug.

Adverse events will be coded according to the Medical Dictionary for Regulatory Activities (MedDRA) adverse event classification. The overall incidence of (serious) adverse events and number and proportion of patients reporting such events will be summarized by treatment group.

Differences in dichotomous outcomes among the two study groups will be analyzed using the chi-squared test or Fisher’s exact test when the expected cell frequencies fall below 5. We will express differences in drug survival as absolute differences and relative risks, with associated 95% confidence intervals, with the group on adalimumab monotherapy as the reference. In case patients are lost to follow-up during the study period, we will analyze these data by means of survival analysis. We will construct cumulative survival curves (Kaplan-Meier method) for the treatment groups, and these curves will be compared using the log-rank test. One-way analysis-of-variance statistics will be calculated to compare continuous outcome measures between groups.

There are no formal planned interim analyses, but progress reports on all data issues are presented to the DSMB.

### Trial status

Patient recruitment started in March 2014 and is currently ongoing. Based on our experience so far, recruitment is limited by two main factors: prior use of adalimumab and intolerability or toxicity for MTX in the past.

Moreover, disease activity in patients who are transitioned from another biologic is often suppressed (<PASI 8). To enlarge the geographical area in which patients can participate in the study and to enhance patient recruitment, three additional hospitals have been activated for patient recruitment: Amphia Hospital (Breda) and Bravis Hospital (Bergen op Zoom) in The Netherlands and Ghent University Hospital in Belgium.

## Discussion

Although combination treatment with anti-TNFs and MTX is being prescribed for psoriasis in clinical practice, available evidence and guidance on the use of combination treatment is limited. No consensus about certain treatment aspects such as timing of initiation of MTX (prior to anti-TNF or during anti-TNF therapy) and MTX dosing exists. Therefore, besides the rationale for our primary endpoints, we would like to emphasize the choice of dosing and initiation of comedication for the current RCT.

### Primary endpoint

In this study, drug survival after 49 weeks of treatment is chosen as the primary endpoint. Based on currently available evidence, response rates to anti-TNFs in patients with and without concomitant MTX may remain similar; however, drug survival is often superior in patients receiving comedication compared to monotherapy, and this difference tends to be more prominent than differences in response rates [33–36]. Moreover, by categorizing reasons for treatment discontinuation (lack of efficacy, safety concerns), several important treatment aspects are being combined.

### Initiation of MTX prior to adalimumab therapy

Concomitant use of MTX has been demonstrated to significantly reduce the clearance of adalimumab, resulting in higher adalimumab trough levels in patients with RA [14, 37]. However, it takes time for MTX to exert a full effect on the pharmacokinetics of adalimumab [37]. The slow onset of drug action of MTX can be attributed to an intracellular accumulation process [38, 39]. After MTX uptake into cells, it is converted to MTX polyglutamates, active metabolites which are believed to exert the anti-inflammatory actions of MTX. The current product label for adalimumab indicates that MTX decreases the apparent clearance of adalimumab after single and multiple doses by 29% and 44%, respectively [37].

In order to ensure maximal potential for MTX to exert a beneficial effect on adalimumab pharmacokinetics from the start on, MTX therapy is initiated 2 weeks before administration of adalimumab in the intervention group.

### Choice of MTX dosing

The dose of MTX as monotherapy can range from 7.5 to 25 mg/week, depending on national guidelines and patient/physician’s preference. A systematic literature review of MTX monotherapy has recommended initial treatment with 10–15 mg orally with dose increases to 20 mg/week if needed and tolerated [40]. Available evidence suggests that MTX toxicity is dose-dependent and low-dose MTX monotherapy treatment can be effective. However, no RCTs have explored the minimally effective dose of MTX in a group of patients when used in combination with a TNFα inhibitor. This dose may differ from minimally effective monotherapy doses.

MTX tends to reduce immunogenicity and increase adalimumab serum levels in a dose-dependent manner in patients with RA. Results indicate a (non-significant) increase in adalimumab serum concentrations with higher doses of MTX (10–20 mg) compared to low-dose MTX. However, a dose of 5–10 mg of concomitant MTX seems already sufficient to substantially decrease immunogenicity against adalimumab and maintain serum concentrations within the therapeutic range [32, 41]. In the treatment of psoriasis, MTX 10 mg per week is an accepted dose for treating psoriasis according to (inter)national guidelines [40]. In order to avoid an increased risk of side effects like hepatotoxicity, a dosage of 10 mg MTX/week is chosen in our RCT over a higher dose.

With this RCT we aim to improve the body of evidence on efficacy and safety of adalimumab and MTX combination treatment in order to investigate whether MTX can optimize adalimumab treatment. Moreover, with the analysis of pharmacogenetic data, we hope to support personalized medicine and more accurate prediction of treatment response.

### Study strengths and limitations

This study represents the first RCT on combined treatment with adalimumab and methotrexate. Data will be extracted and analyzed independent of industry. It is an observer-blinded study with concealment of allocation. Clinical as well as pharmacokinetic and pharmacogenetic outcomes will be assessed in the short and long term.

However, some limitations apply. Due to the pragmatic study design, the trial is not conducted as double-blind. Moreover, the sample size limits assessment of the predictive performance of genetic polymorphisms on clinical and pharmacokinetic outcomes. Optimal dosing and timing of MTX comedication are not evaluated in this study and will have to be investigated in future research.

## Additional file

Additional file 1: SPIRIT 2013 checklist: recommended items to address in a clinical trial protocol and related documents.* (DOC 121 kb)

## Abbreviations

DLQI: Dermatology Life Quality Index
DSMB: Data Safety Monitoring Board
MTX: Methotrexate
PASI: Psoriasis Area and Severity Index
RA: Rheumatoid arthritis
RCT: Randomized controlled trial
SNP: Single nucleotide polymorphism
TNF: Tumor necrosis factor

## Footnotes

1 Treatment goals will be achieved if patients reach PASI ≥ 75 or PASI ≥ 50 in combination with DLQI ≤ 5. Treatment goals will not be achieved if PASI < 50 or PASI ≥50 < 75 in combination with DLQI ≥ 5 [42].
