# Erratum to: Optimizing adalimumab treatment in psoriasis with concomitant methotrexate (OPTIMAP): study protocol for a pragmatic, single-blinded, investigator-initiated randomized controlled trial

**DOI:** 10.1186/s13063-017-1848-0

**Published:** 2017-03-07

**Authors:** Celine Busard, Stef Menting, Sun-Jine van Bezooijen, Juul van den Reek, Barbara Hutten, Errol Prens, Elke de Jong, Martijn van Doorn, Phyllis Spuls

**Affiliations:** 1 Department of Dermatology, Academic Medical Center, Amsterdam, The Netherlands; 2 Department of Dermatology, Erasmus University Medical Center, Rotterdam, The Netherlands; 3 Department of Dermatology, Radboud University Medical Center, Nijmegen, The Netherlands; 4 Department of Clinical Epidemiology, Biostatistics and Bioinformatics, Academic Medical Center, Amsterdam, The Netherlands

## Erratum

The author initials were not complete in the original publication [1].

The correct initials should be: “CI Busard, SP Menting, JS van Bezooijen, JM van den Reek, BA Hutten, EP Prens, EM de Jong, MB van Doorn, PI Spuls”.

## References

[1] Optimizing adalimumab treatment in psoriasis with concomitant methotrexate (OPTIMAP): study protocol for a pragmatic, single-blinded, investigator-initiated randomized controlled trial. Trials. 2017;18:52 doi: 10.1186/s13063-017-1777-y. 10.1186/s13063-017-1777-y PMC5288945 28148280

